# A Miniature Bioassay for Testing the Acute Phytotoxicity of Photosystem II Herbicides on Seagrass

**DOI:** 10.1371/journal.pone.0117541

**Published:** 2015-02-12

**Authors:** Adam D. Wilkinson, Catherine J. Collier, Florita Flores, Phil Mercurio, Jake O’Brien, Peter J. Ralph, Andrew P. Negri

**Affiliations:** 1 College of Marine and Environmental Sciences, James Cook University, Townsville, Queensland, Australia; 2 Australian Institute of Marine Science, Townsville, Queensland, Australia; 3 Centre for Tropical Water & Aquatic Ecosystem Research (TropWATER), James Cook University, Cairns, Queensland, Australia; 4 School of Medicine, University of Queensland and National Research Centre for Environmental Toxicology, Coopers Plains, Queensland, Australia; 5 Plant Functional Biology and Climate Change Cluster (C3), University of Technology, Sydney, New South Wales, Australia; University of Hyderabad, INDIA

## Abstract

Photosystem II (PSII) herbicides have been detected in nearshore tropical waters such as those of the Great Barrier Reef and may add to the pressure posed by runoff containing sediments and nutrients to threatened seagrass habitats. There is a growing number of studies into the potential effects of herbicides on seagrass, generally using large experimental setups with potted plants. Here we describe the successful development of an acute 12-well plate phytotoxicity assay for the PSII herbicide Diuron using isolated *Halophila ovalis* leaves. Fluorescence images demonstrated Diuron affected the entire leaf surface evenly and responses were not influenced by isolating leaves from the plant. The optimum exposure duration was 24 h, by which time the inhibition of effective quantum yield of PSII (∆F/F_m_’) was highest and no deterioration of photosystems was evident in control leaves. The inhibition of ∆F/F_m_’ by Diuron in isolated *H. ovalis* leaves was identical to both potted and hydroponically grown plants (with leaves remaining attached to rhizomes), indicating similar reductions in photosynthetic activity in these acute well-plate assays. The sensitivity of the assay was not influenced by irradiance (range tested 40 to 400 μmol photons m^-2^ s^-1^). High irradiance, however, caused photo-oxidative stress in *H. ovalis* and this generally impacted in an additive or sub-additive way with Diuron to damage PSII. The bioassay using isolated leaves is more rapid, uses far less biological material and does not rely on specialised aquarium facilities in comparison with assays using potted plants. The development and validation of this sensitive bioassay will be useful to reliably screen and monitor the phytotoxicity of existing and emerging PSII herbicides and contribute to risk assessments and water quality guideline development in the future.

## Introduction

### Tropical seagrass significance

Seagrasses provide essential nursery and feeding grounds for commercially important fish, crustaceans and molluscs and form, almost exclusively, the diet of a number of macro-grazers, including the endangered dugong and green sea turtles [1]. Seagrass also promotes sediment stabilisation [1,2], nutrient cycling [3] and carbon sequestration [4]. As far back as two decades ago, the net total ecosystem services that seagrass provide annually were estimated to be worth $3.8 trillion [5]. However, seagrass habitats are declining globally with approximately 110 km^2^ lost annually [6,7].

### Effects of water quality on seagrass populations

The degradation of water quality factors in the dramatic decline of seagrass meadows around the globe [1]. One of the best-studied examples is the Great Barrier Reef (GBR) which experiences heavy rainfall over the summer wet season, delivering large amounts of suspended solids into near shore habitats, resulting in reductions in light for primary productivity by benthic species [8]. Seagrass loss on the GBR is strongly influenced by long periods of severe light attenuation caused by suspended solids and phytoplankton that peak during these flood events [9]. Along with increased sediments and nutrients, several agricultural herbicides which target photosystem II (PSII) have been regularly detected in the catchment rivers and estuaries and within nearshore habitats of the GBR lagoon [10,11,12].

### Herbicides and seagrass

Diuron is one of the most commonly detected PSII herbicides in inshore and coastal waters adjacent to the GBR [12,13,14]. Diuron has been detected year-round in the GBR though concentrations peak during the wet season when flood plumes deliver herbicides that are washed from the land into waterways during heavy rainfall [15,16]. Upon delivery to the GBR lagoon, some dilution of herbicides occurs and yet PSII herbicides have also been detected within river plumes at concentrations up to 1 μg l^-1^ [12]. Diuron has been sampled in sub-tidal sediments (up to 10 μg kg^-1^), within intertidal seagrass specimens (up to 1.7 μg kg^-1^) [17] and in creeks flowing into the GBR lagoon (up to 8.5 μg l^-1^) [13]. PSII herbicides block electron transport in PSII and a Diuron concentration of 0.5 μg l^-1^ reduces photosynthetic efficiency by 10% in two GBR species [18]. Longer-term effects from chronic photoinhibition include plant starvation, reduced growth and finally adverse effects on competitive fitness, likely impacting on higher trophic levels [10,19,20].

### Acute seagrass assay development

A highly relevant and sensitive indicator of PSII herbicide effects on seagrasses and other photosynthetic organisms such as corals, is the change in quantum yield of PSII as measured using pulse amplitude modulated (PAM) fluorometers [19,21]. The most sensitive parameter measured by PAM fluorometers is inhibition of effective quantum yield (*∆F/F*_*m*_’) [18,19]. This is proportional to reduced photosynthetic efficiency under experimental light levels and provides a link to diminished photosynthetic carbon fixation (energy) [19,22]. PAM fluorometry has also been used to measure inhibition of the maximum quantum yield (*F*_v_/*F*_m_) which is proportional to damage to PSII due to oxidative stress caused by PSII herbicides blocking electron transport under illuminated conditions [22,23]. Plotting inhibition of quantum yields against PSII herbicide concentrations yields typical dose-response curves from which herbicide concentrations that inhibit 10 and 50% of *∆F/F*_*m*_’ and *F*_v_/*F*_m_ (IC_10_ and IC_50_) can be derived [18].

Previous studies have used PAM fluorometers with flexible fibre optic probes to measure fluorescence responses of individual leaves from intact potted plants exposed to herbicides for ~3 days [10,18,24]. While providing precise and relevant ecotoxicological data, the whole-plant assays require large experimental aquarium setups and many days of preparation and maintenance. A potentially rapid and sensitive alternative approach is to apply the spatial imaging Maxi-Imaging-PAM (I-PAM) (Walz, GmbH Germany) to individual seagrass leaves in well-plates. This approach has been successfully used to quantify the toxicity of PSII herbicides on isolated whirls and fronds of multiple macroalgae species in 24-well [25], and in 96-well formats with several species of microalgae [26,27]. The application of I-PAM to small individual seagrass leaves offers the potential of developing a more rapid and less expensive method to assess and monitor the phytotoxicity of PSII herbicides to seagrass under a range of environmental conditions.

In this study, we quantified the acute phytotoxicity of PSII herbicide, Diuron, on *Halophila ovalis* while validating a 12-well plate fluorescence bioassay using the I-PAM. Fluorescence-derived phytotoxicity endpoints in the isolated leaves were directly compared with potted and unpotted but intact (hydroponic) seagrasses and the influence of light on photosynthetic efficiency and damage to PSII were assessed.

## Materials and Methods

A miniature 12-well plate phytotoxicity assay was developed to assess the exposure of seagrass to PSII herbicides in the following way:-

1. Sample collection and experimental setup. All acute exposures (up to 24 h) were conducted in static conditions using measured concentrations of Diuron.
2. PAM fluorometry (see below) was applied as a specific and sensitive indicator of PSII herbicide toxicity to isolated seagrass leaves and intact plants.
3. Leaves were screened for high levels of photosynthetic efficiency before the start of each experiment.
4. Rapid light curves were used to assess the photosynthetic performance of the seagrass as a function of irradiance and to enable the selection of ambient illumination for the experiments.
5. Fluorescence images were captured using the I-PAM to spatially monitor photosynthetic impact of Diuron in the isolated leaves.
6. The photosynthetic condition of leaves were re-examined by I-PAM regularly over 24 h in the absence of herbicide to test for leaf deterioration over the exposure period. The impact of Diuron on effective quantum yields was also monitored to ensure maximum uptake (a steady state of toxic effect) had been reached within 24 h.
7. Dose-response relationships were compared between I-PAM and Mini-PAM data to verify consistency with other studies.
8. Dose-response relationships were compared between isolated leaves in 12-well plates and intact plants (both potted and hydroponic) to validate the sensitivity of the well-plate method.
9. Dose-response relationships were compared using the well plate method at four light levels to (i) test consistency and repeatability under different irradiance conditions and (ii) examine the potential for Diuron to impact on seagrass under varying light conditions.
10. Potential interactions between irradiance and Diuron on effective and maximum quantum yields were explored using the Independent Action (IA) model.

### Sample collection and experimental setup (1)

*Halophila ovalis* is a tropical seagrass species widely distributed throughout the Indo-Pacific and is found in all marine habitats throughout Australia [28] and is one of the target species for *Dugong dugon* foraging [29]. It is a rapidly growing species with leaf pairs emerging from the rhizome. It is also highly sensitive to environmental stress [24,30,31,32], responding like an r-strategist by dying off when stressed and re-populating from its seedbank [33]. Its rapid response time to habitat modification could make it an excellent sentinel species, providing early warning of changes to environmental conditions. *H*. *ovalis* plants were collected from intertidal meadows during low tide from Cockle Bay, Magnetic Island (19° 10.88’S, 146° 50.63’E) under permit MTB41, a permit issued for limited impact research in the GBR Marine Park which was assessed through the Department of Employment, Economic Development and Innovation self-assessable Fisheries Queensland Code MP05 for the removal of marine plants.

A small plug of seagrass with its associated sediment (5–10 cm depth) was removed and placed in plastic plant pots lined with plastic bags. The bag was pulled up over the seagrass and a small amount of water was added to the bag and secured at the top for transport. Plants were taken to the Australian Institute of Marine Science (AIMS) and placed into 1000 l outdoor aquaria within 4 h from collection under 20% ambient light (maximum of ~ 400 μmol photons m^-2^s^-1^ under shade cloth) and water temperature conditions (25–28°C).

Diuron, also called DCMU or (3-(3,4-dichlorophenyl)-1,1-dimethylurea) is a phenyl-urea group photosystem II herbicide and was obtained from Sigma Aldrich (>95% pure). Stock solutions of 5 mg l^-1^ Diuron in milli-Q water were prepared using 2 ml ethanol (<0.03% v/v solvent carrier in exposures). Individual working solutions of each concentration were then prepared in 0.45 μm-filtered seawater with the same proportion of ethanol carrier. Water samples for herbicide analysis (2 ml) were taken 1 h and 24 h after dosing and pipetted into 4 ml amber glass vials. Samples were then spiked with 20 μl of a surrogate standard, d5-Atrazine (Novachem, Victoria, Australia) and stored frozen. Diuron was analysed using LC-MS/MS as per Flores et al. [18].

### PAM fluorometry (2)

Pulse Amplitude Modulated (PAM) fluorometer measurements were conducted using two different instruments for comparison. Mini-PAM measurements were obtained by placing a 2 mm fibre optic probe perpendicular to the leaf surface, approximately at the centre, offset from the mid-vein. Measurements were made only on young, healthy leaves (second or third leaf pair from the terminal end of the rhizome). Minimal fluorescence (*F* with illuminated samples and *F*_*0*_ with dark-adapted samples) was determined by applying a weak pulse-modulated red measuring light (650 nm, 0.15 μmol photons m^-2^s^-1^). To quantify light adapted maximum fluorescence (*F*’_*m*_), a short pulse (800 ms) of saturating actinic light (>3000 μmol photons m^-2^s^-1^) was applied and the effective quantum yield of PSII calculated from *∆F/F*_*m*_′ = (*F*_*m*_′—*F*) / *F*_*m*_′. In order to calculate the maximum quantum yield of PSII (*F*_v_/*F*_m_), seagrass was dark adapted for 30 min and *F*_*0*_ and *F*_*m*_ measured, as above, from *F*_*v*_/*F*_*m*_ = (*F*_*m*_—*F*_*0*_) / *F*_*m*_. *F*_v_/*F*_m_ is a measure of the optimal photosynthetic efficiency and inhibition of *F*_v_/*F*_m_ can indicate photooxidative stress and damage to PSII from irradiance stress. *∆F/F*_*m*_′, on the other hand, reflects the level of PSII activity under ambient light conditions, providing a more sensitive and realistic assessment of PSII herbicide impacts on photosynthesis [19].

Imaging-PAM (I-PAM) measurements were conducted in 12-well plates by individually placing each well plate into the imaging chamber and controlled using Data- MAXI software on a desktop computer (Imaging Win, Walz GmbH, Germany). Actinic light was set to 100 μmol photons m^-2^s^-1^ and a measuring intensity of four was applied to generate similar quantum yields as observed for Mini-PAM measurements. Following the imaging process, Imaging Win was used to select single area of interest (AOI) of 3–5 mm diameter for each leaf in order to maximise the leaf surface area for yield measurements. *ΔF/F*_m_’, and *F*_v_/*F*_m_, were calculated as above and were typically measured prior to dosing and at 24 h (end of exposure).

### Seagrass leaf screening and general bioassay conditions (3)

Before removing leaves, any epiphyte growth was removed from the leaf surface. Stems were cut to the base of the leaf and pinched closed with forceps to minimise formation of air bubbles in the midrib. Any bubbles on the external surface of the leaf were gently removed to prevent floating. Before each I-PAM measurement, all leaves which were not horizontally orientated were repositioned until flat against the bottom of the well. Care was taken to ensure the leaf was not in contact with the well wall to minimise fluorescence interference. Once in the I-PAM chamber a ~5 sec interval was taken to ensure all water movement ceased before measurements were taken.

To ensure only healthy, reliable leaves were used in the well plate experiments, cut leaves underwent a screening process. Second and third leaf pairs from the terminal, apical end of the rhizome were selected and removed. Single leaves were placed in each well of 12-well plates (Nunclon, Thermo scientific) containing 0.45 μm filtered seawater (5 ml each well). Maximum quantum yield of each cut leaf was measured using the I-PAM and only leaves exhibiting *F*_*v*_/*F*_*m*_ greater than 0.65 were used to ensure optimal leaf health and to allow for greater reliability. This threshold was chosen based on greater consistency of measurements in leaves taken over 24 h in our pilot experiments when initial *F*_*v*_/*F*_*m*_ was greater than 0.65. Average leaf length was 10.0 mm ± 2.5 (range of all leaves) and width was 4.8 mm ± 1.2. This screening process was performed immediately prior to running the assays to ensure the leaves were in healthy condition for the experiment.

### Irradiance conditions (4)

Prior to experiments, the plants were maintained for at least 48 h upright in aquaria under a constant temperature of 26°C ± 1, salinity of 34–36 ppt and light of 280 ± 15 (range under all lights) μmol photons m^-2^s^-1^ (Aqua Illumination SOL White LED lights) which was equivalent to the mean irradiance at the collection site from 2008–2012 (Collier unpub.). For well-plate assays, leaves were placed horizontally in filtered seawater and left for at least 1 hr at 100 μmol photons m^-2^s^-1^. The effective quantum yield of PSII (*ΔF/F*_*m*_’) were equivalent (~ 0.60–0.65) for vertical leaves at 280 μmol photons m^-2^s^-1^ and horizontal leaves at 100 μmol photons m^-2^s^-1^ using the same light source, indicating that the proportion of PSII reaction centres closed due to photosynthetic activity was equivalent under both conditions (horizontal leaves were almost 3-fold more photosynthetically active due to greater light interception).

In order to assess the photosynthetic performance of the seagrass as a function of irradiance, rapid light curves (RLC) were used with the I-PAM for leaves in well plates maintained for > 1 h under 100 μmol photons m^-2^s^-1^ [34]. *∆F/F*_*m*_′ was measured using the I-PAM software at 12 actinic light intensities and the relative electron transport rate (rETR) was calculated and plotted against irradiance according to the equation rETR = *∆F/F*_*m*_′ PAR 0.5 [34]. Minimum saturating irradiance (*E*_k_) and maximum photosynthetic capacity (rETR_max_) were also calculated.

### Spatial response of seagrass to herbicide uptake (5)

To assess the homogeneity of Diuron uptake in isolated *H*. *ovalis* leaves, the fluorescence response to 10 μg l^-1^ Diuron was observed using I-PAM. Leaves were acclimated under 100 μmol photons m^-2^ s^-1^ at 27°C for 2 h in 12-well plates with 5 ml filtered seawater. Leaves were individually exposed to 10 μg l^-1^ Diuron and a solvent control and Δ*F/F*_*m*_’ and *F*_*v*_/*F*_*m*_ measured over 24 h.

### Exposure durations (6)

The duration of acute exposure for the well-plate assay needed to be long enough to ensure steady-state (maximum) inhibition of Δ*F/F*_m_’ in *H*. *ovalis* but short enough so that the leaves had not appreciably deteriorated (no substantial decline in photosynthetic capacity *F*_v_/*F*_m_’ in controls). Leaves were placed in 12-well plates and acclimated horizontally at 100 μmol photons m^-2^ s^-1^. Leaves (n = 16) were exposed to 10 μg l^-1^ Diuron or solvent control solutions for up to 24 h. Δ*F/F*_m_’ was measured at 0, 2, 4, 6, 12 and 24 h and *F*_v_/*F*_m_’ at 0, 12 and 24 h. All subsequent bioassay experiments were performed over 24 hours when >95% of maximum inhibition had been reached at 10 μg l^-1^ Diuron.

### Dose-responses from I-PAM and Mini-PAM (7)

Dose response curves were produced for the inhibition of Δ*F/F*_m’_ and *F*_v_/*F*_m_ in response to seven elevated Diuron concentrations in well-plate assays under an illumination of 100 ± 10 μmol photons m^-2^s^-1^. Single leaves, with all stems removed, were placed within each individual well and allowed to acclimate for ~2 h. Leaves were then transferred to 12-well plates so each well plate contained three leaf replicates of the solvent control and three Diuron concentrations (i.e. four treatments three replicate = 12 wells). Concentrations were randomised across all well plates to minimise any well plate effect. There were a total of nine replicate leaves for each Diuron concentration across multiple plates. The % inhibition of Δ*F/F*_m_’ and *F*_v_/*F*_m_ relative to solvent control treatments were calculated and plotted as dose-response curves (see below). The mean measured concentrations used in each dose-response relationship within this study were: 0 (below reporting limit 0.05), 0.08, 0.24, 0.80, 2.2, 8.2, 31 and 113 μg l^-1^. In order to examine potential differences in Diuron phytotoxicity to *H*. *ovalis* leaves calculated using Mini-PAM and I-PAM measurements, a direct comparison of dose-responses was conducted following the 24 h Diuron exposure period.

Photoinhibition (inhibition % relative to solvent control) was calculated from treatment data as Inhibition (%) = [(Y_control_-Y_sample_)/Y_control_] 100, where Y is Δ*F/F*_m_’ or *F*_v/_ *F*_m_. A dose response curve was plotted using inhibition data at 24 h exposure. A four parameter logistic curve was fitted to each data set separately (Sigmaplot 11.0) [18]. IC_50_ values and corresponding confidence intervals were determined from each curve by applying standard curve analysis. The probability that midpoints (IC_50_s) generated by the logistic curves were statistically different was tested by applying the *F* test in Graph Pad Prism V 6.0. IC_50_s were considered different when *p* < 0.05 and the post-hoc results presented for each comparison in the relevant Results sections.

### Response of single leaf well plate vs hydroponic and potted plants (8)

The photobiological response of single leaves to Diuron in 12-well plate format exposures was compared with intact potted plants (including sediment to 4.5 cm depth) and hydroponic plants (whole plants in 0.45 μm filtered seawater without sediments or nutrients). While potted plants most closely represent the natural state, hydroponic plants represent an intermediate or bare state where the entire root-rhizome system is intact but not submerged in sediments. Potted plant samples were contained in 500 ml experimental pots (13.5 9.8 4.5 cm). The hydroponic and potted arrangements were placed in 6 l indoor glass aquaria [18]. A constant temperature of 26°C ± 1, salinity of 34–36 and light of 280 ± 15 (range) μmol photons m^-2^s^-1^ was maintained. Single leaf well plate exposures were performed as described above.

### Effects of irradiance on assay sensitivity and damage to PSII (9)

To assess how irradiance and Diuron affected the photosynthetic efficiency of *H*. *ovalis*, the seagrass was exposed to 40, 100, 200 and 400 μmol photons m^-2^ s^-1^ with varying concentrations of Diuron (0–100 μg l^-1^). This experiment utilised the well plate bioassay with isolated horizontal leaves and dose-response curves for inhibition of effective and maximum fluorescence yields were developed. Inhibition was calculated in two ways: (i) in comparison to the yield of the solvent control at the relevant irradiance and (ii) in comparison to the yield of the solvent control at the lowest irradiance.

### Exploring interactions between irradiance and Diuron on photosynthetic yields (10)

Expected inhibition of both Δ*F/F*_m_’ and *F*_v/_ *F*_m_ for additivity of effects was calculated by applying the Independent Acion (IA) equation to the inhibition data: P(L, D)_p_ = P(L) + P(D) – P(L) × P(D) [35,36]. Here P(L, D)_p_ is the predicted additive effect of both variables tested; P(L) is the effect of light intensity in the absence of Diuron and P(D) is the effect of Diuron at the control light level, 40 μmol photons m^-2^ s^-1^. Both P(L) and P(D) are derived from raw data means. Expected combined effect of both light intensity and Diuron exposure were calculated for seven herbicide and four light combinations and plotted against the measured inhibition data relative to the 40 μmol photons m^-2^ s^-1^ light intensity treatment solvent control (greatest yields of all four light treatments).

## Results

### Leaf screening and general bioassay conditions (1–3)

A series of preliminary experiments indicated that consistent maximum quantum yield measurements were only possible following careful pre-screening of the leaves (initial *F*_*v*_/*F*_*m*_ greater than 0.65). Damaged leaves and/or those exhibiting low *F*_*v*_/*F*_*m*_ values deteriorated over 24 h in well plates resulting in low and often erratic fluorescence response.

### Irradiance conditions (4)

A rapid increase in rETR was observed during the light limiting region of the curve (α = 0.43). The minimum saturating irradiance (*E*_k_) of 44 μmol photons m^-2^ s^-1^ was relatively low, indicating that the seagrass maintained in the indoor aquarium at 280 μmol photons m^-2^s^-1^ for upright leaves followed by horizontal acclimation for the assay at 100 μmol photons m^-2^s^-1^ were adapted to relatively low light. The rETR reached a maximum of 19 μmol electrons m^-2^s^-1^. These measurements allowed us to choose four irradiances for subsequent experiments that represented light limiting (40 μmol photons m^-2^ s^-1^), saturated (100 μmol photons m^-2^ s^-1^) and two higher irradiances representing irradiance-stressed conditions at 200 and 400 μmol photons m^-2^ s^-1^ where illumination exceeded the photosynthetic capacity of the seagrass (leading to elevated photoinhibition).

### Spatial response of seagrass to herbicide uptake (5)

**Fig 1:**
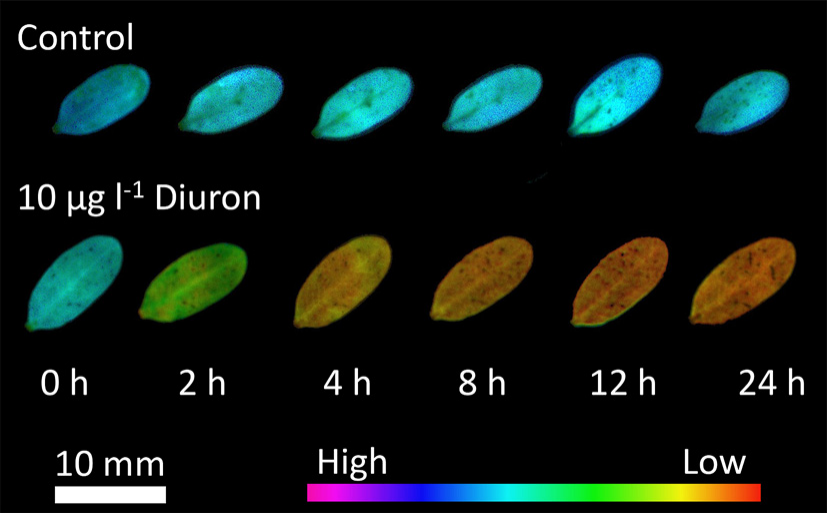
Series of images. ∆*F*/*F*_m_’ measured using an I-PAM over a 24 h period. One series illustrates a solvent control leaf over 24 h while the other illustrates changes in ∆*F*/*F*_m_’ due to exposure to 10 μg l^-1^ Diuron over the same period.

The isolated *H*. *ovalis* leaves exhibited an even change in fluorescence across their surface following exposure to Diuron (Fig. 1). There was a small area near the cut stem that showed reduced ∆*F*/*F*_m_’, but Diuron affected the entire surface of the leaves rather than spreading from the stem and the midrib (the stem was therefore not chosen as part of the area of interest for any quantitative experiments).

### Time taken to reach maximum toxicity (6)

**Fig 2:**
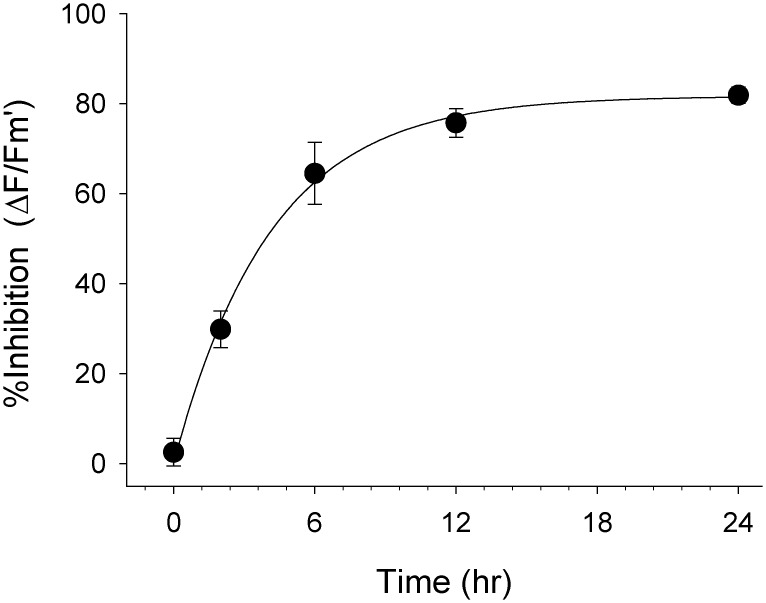
Time taken to reach steady state toxicity. Inhibition of effective quantum yield (*ΔF/F*_m_’) in *H*. *ovalis* by 10 μg l^-1^ Diuron relative to solvent control conditions versus time. Bars ± SE, n = 16.

Leaves selected by pre-screening (above) exhibited little reduction in *F*_v_/*F*_m_ from 0.71 ± 0.01 (SE) to 0.69 ± 0.01 (less than 3%) in solvent controls indicating limited deterioration or additional photoinhibitory stress over 24 h. In this experiment 10 μg l^-1^ Diuron inhibited Δ*F/F*_m_’ by 82% ± 2% (SE) after 24 h relative to solvent control leaves. The uptake of Diuron was rapid with 95% maximum inhibition of Δ*F/F*_m_’ (toxicity) reached after 12 h and steady state toxicity between 18 and 24 h (Fig. 2), indicating that consistent toxicity should be expected between experiments after exposure periods of 18 h or more.

### Dose-responses from I-PAM and Mini-PAM (7)

**Fig 3:**
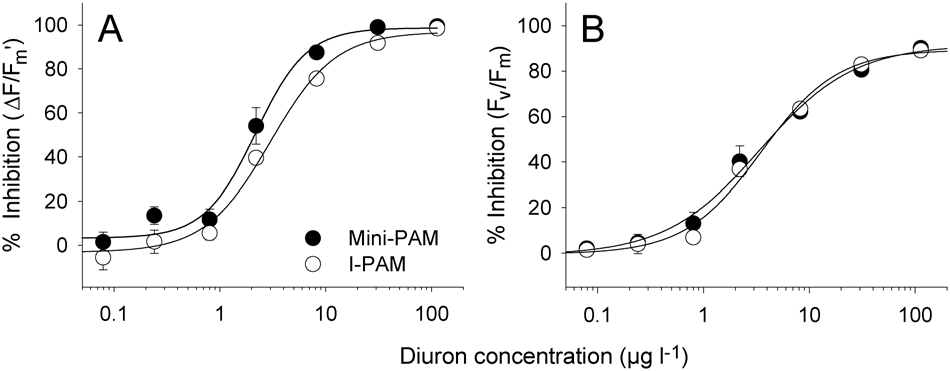
Dose-response curves for Diuron and *H*. *ovalis* using different PAM fluorometers. Inhibition (%, relative to solvent control) of A) effective quantum yield (∆*F*/*F*_m’_) and B) maximum quantum yield *(F*_v_/*F*_m_) of the single leaf, multi-well plate sample orientation. Results from both I-PAM and Mini-PAM following a 24 h exposure period to a series of Diuron concentrations at 100 μmol photons m^-2^s^-1^. Mean ± SE of nine replicate leaves. Curves exhibited r^2^ between 0.98 and 0.99.

Dose-response curves obtained from different PAM fluorometers indicated that although the inhibition of *ΔF/F*_m_’ by Diuron was similar in each case (Fig. 3A) that the mini-PAM was slightly but significantly (*F*_3, 24_ = 5.40, *p* < 0.05) more sensitive to Diuron. The Diuron concentrations that inhibited ∆*F*/*F*_m_’ by 10% and 50% (IC_10_ and IC_50_) can be found in Table 1. Inhibition of *F*_v_/*F*_m_ in *H*. *ovalis* by Diuron was consistent between techniques (Fig. 3B and Table 1, *F*_3, 24_ = 27.4, *p* > 0.05). The complete inhibition of *F*_v_/*F*_m_ was not reached over this range of exposures potentially due to incomplete binding of Diuron to the binding sites of PSII.

**Table 1:** Diuron concentrations that inhibit 50% (IC_50_) and 10% (IC_10_) quantum yields in different sample arrangements of *H*. *ovalis*.

	∆*F*/*F*_m_’				*F*_v_/*F*_m_			
	IC_50_	95% CV	IC_10_	95% CV	IC_50_	95% CV	IC_10_	95% CV
12-well I-PAM	3.5^a^	2.5–4.3	0.78	0.46–1.2	4.3^a^	3.1–6.3	0.66	0.33–1.14
12-well Mini-PAM	2.1^b^	1.5–3.2	0.53	0–1.1	4.2^a^	3.1–5.9	0.44	0.20–0.75
Potted Mini-PAM	3.0^a^	2.6–3.5	0.79	0.59–1.0	12^b^	7–24	0.86	0.27–2.1
Hydroponic Mini-PAM	3.5^a^	3.0–4.0	1.0	0.81–1.3	16^b^	12–22	1.5	1.1–2.1

Different superscripted letters indicate statistically different IC_50_ values (p < 0.05).

### Dose-response relationships for 12-well plates in comparison with potted and hydroponic plants (8)

**Fig 4:**
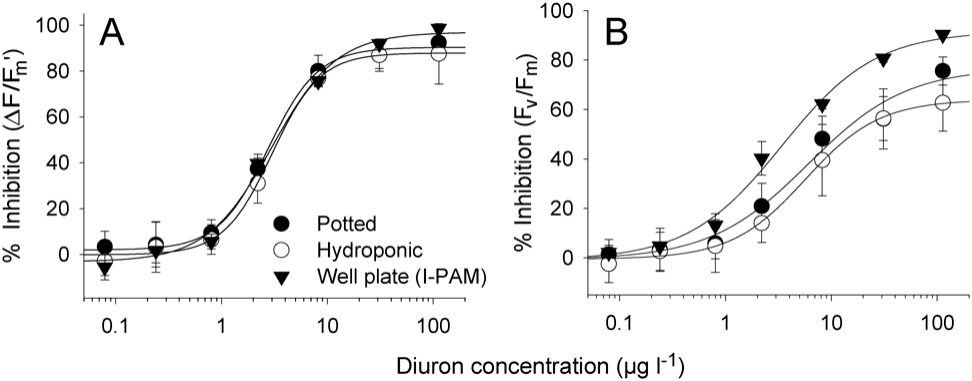
Dose-response curves for Diuron and *H*. *ovalis* using 12-well plate vs potted and hydroponic arrangements. Inhibition (%) of A) effective quantum yield (∆*F*/*F*_m_’) and B) maximum quantum yield of PSII (*F*_v_/*F*_m_) after a 24 h exposure period to a series of Diuron concentrations at 100 μmol photons m^-2^s^-1^. Yield inhibitions were calculated against mean yield values for solvent controls at the corresponding irradiance. n = 9 ± SE, curve fits exhibited r^2^ between 0.98 and 0.99.

The effect of Diuron on ∆*F*/*F*_m_’ in *H*. *ovalis* leaves in 12-well plates was identical to whole potted or hydroponic plants, with IC_10_ values ranging from 0.53 to 1.0 and IC_50_ values ranging from 3.0 to 3.5 μg l^-1^ (Fig. 4A and Table 1, *F*_3, 24_ = 5.40, *p* > 0.05). However, Diuron caused more inhibition of *F*_v_/*F*_m_ in well plate leaves which exhibited IC_50_ for *F*_v_/*F*_m_ of 4.2 μg l^-1^ compared with 11 and 16 μg l^-1^ for potted and hydroponic arrangements respectively (Fig. 4B, Table 1, *F*_3, 24_ = 27.4, *p* < 0.05).

### Effects of irradiance on assay sensitivity (9)

**Fig 5:**
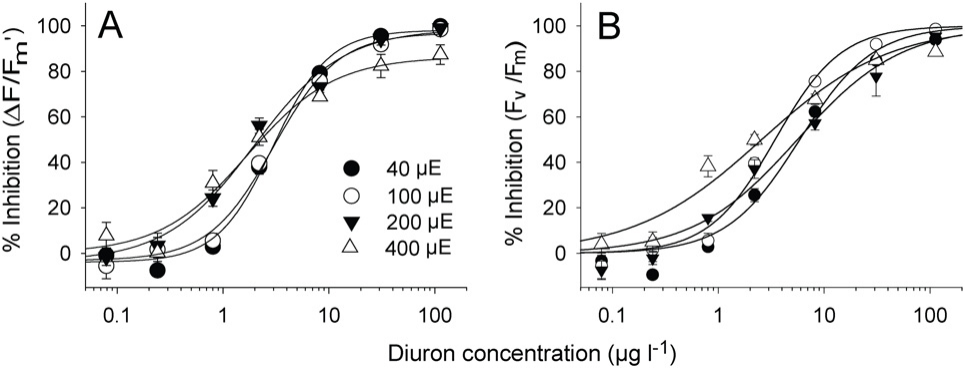
Dose-response curves for Diuron and *H*. *ovalis* at four different irradiances relative to the yields of corresponding irradiances. Inhibition (%) of A) effective quantum yield (∆*F*/*F*_m_’) and B) maximum quantum yield of PSII (*F*_v_/*F*_m_) after a 24 h exposure to a series of Diuron concentrations at four irradiances. Yield inhibitions were calculated against mean yield values for solvent controls at the corresponding irradiance. Mean ± SE of nine replicate leaves. All curves fits exhibited r^2^ between 0.98 and 0.99.

The inhibition of ∆*F*/*F*_m’_ relative to solvent controls at the corresponding light intensity was relatively consistent across herbicide exposures (Fig. 5A). Although the IC_10_s and IC_50_s at the 200 and 400 μmol photons m^-2^ s^-1^ were numerically lower than those under lower illuminations, the differences were not significant (Table 2, *F*_3, 24_ = 2.00, *p* > 0.05). Similar results were observed for inhibition of *F*_v_/*F*_m_ by Diuron when measured against the controls at corresponding light intensities (Fig. 5B, Table 2), although the IC_50_ for *H*. *ovalis* exposed at 400 μmol photons m^-2^ s^-1^ was lower than for the other treatments (F_3, 24_ = 5.83, p < 0.05).

**Table 2:** Diuron concentrations (g l^-1^) that inhibit 50% (IC_50_) and 10% (IC_10_) of *H*. *ovalis* leaf quantum yields at different irradiance levels.

	∆*F*/*F*_m’_				F_v_/F_m_			
Irradiance	IC_50_	95% CV	IC_10_	95% CV	IC_50_	95% CV	IC_10_	95% CV
40	3.3^a^	2.4–4.5	0.97	0.57–1.53	5.4^a^	3.9–8.0	1.23	0.67–1.97
100	3.5^a^	2.5–4.3	0.78	0.46–1.27	4.3^a^	3.1–6.3	0.66	0.33–1.14
200	2.2^a^	1.5–3.5	0.32	0.13–0.64	5.3^a^	3.1–9.8	0.55	0.19–1.22
400	2.3^a^	1.2–4.6	0.25	0.07–1.03	2.7^b^	1.3–4.4	0.18	0.06–0.49

Yield inhibitions were calculated against mean yield values for solvent controls at the corresponding irradiance (μmol photons m^-2^ s^-1^). Different superscripted letters indicate statistically different IC_50_ values (p < 0.05).

**Fig 6:**
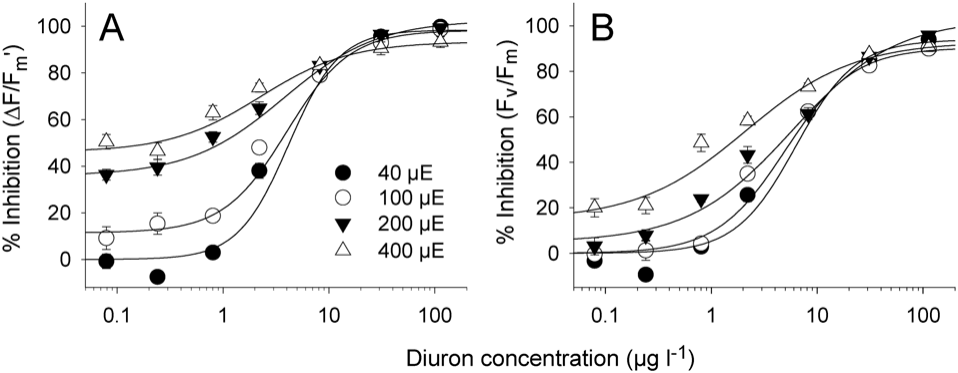
Dose-response curves for Diuron and *H*. *ovalis* at four different irradiances relative to the control yields held under 40 μmol photons m^-2^ s^-1^. Inhibition (%) of A) effective quantum yield (∆*F*/*F*_m_’) and B) maximum quantum yield of PSII (*F*_v_/*F*_m_) after a 24 h exposure period to a series of Diuron concentrations at four irradiances. Yield inhibitions were calculated against mean yield values for solvent controls at the lowest irradiance. Mean ± SE of nine replicate leaves. All curves fits exhibited r^2^ between 0.97 and 0.99.

The combined reduction of ∆*F*/*F*_m_’ relative to the solvent control measurements was far greater under increased illumination compared to those taken under 40 μmol photons m^-2^ s^-1^ (Fig. 6A). At low Diuron concentrations there was a clear step-wise inhibition that increased with light due to the increased photosynthetic activity. The inhibition converged at approximately 80% when the Diuron concentration reached 10 μg l^-1^.

### Fitting the effects of irradiance and Diuron to the Independent Action model (10)

**Fig 7:**
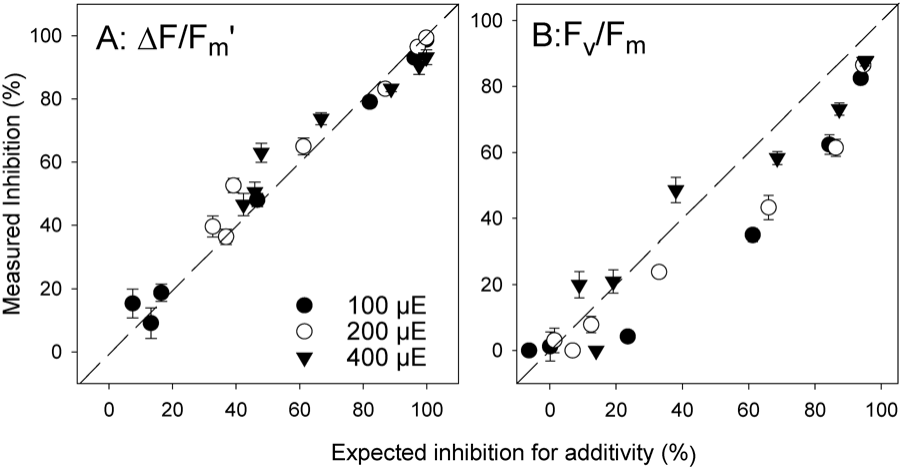
Measured vs expected toxicities. Comparison between measured and expected (IA) combined effects of elevated light intensities and Diuron on A) ∆*F*/*F*_m’_ and B) *F*_v_/*F*_m_ of *H*. *ovalis*. Data points intersecting the zero-interaction line indicate additivity; points below the additivity line suggest sub-additivity; and datapoints above the additivity line indicate synergism. All inhibition calculated relative to 40 μmol photons m^-2^ s^-1^ solvent control mean. Mean ± SE of nine replicate leaves.

The mixture model of Independent Action (IA) was applied to ∆*F*/*F*_m_’ inhibition to test whether the effects of light and Diuron were additive, synergistic or sub-additive. A plot of expected combined inhibitions (IA) against measured inhibitions (Fig. 7A) revealed a strong agreement with the additive model (most of the data points overlapped the 1:1 line indicating additivity).

A similar series of converging dose-response curves were observed for inhibition of *F*_v_/*F*_m_ at four light intensities and seven Diuron concentrations (Fig. 6B). An inhibition of *F*_v_/*F*_m_ of ~20% was observed under 400 μmol photons m^-2^ s^-1^ at low Diuron concentrations indicating light-induced damage to photosystem II at this irradiance. For each combination of light pressure and Diuron concentration, inhibition increased. The IA plots of expected vs observed inhibition for combined herbicide and light stress (Fig. 7B) indicated additivity of these pressures (data overlapped the 1:1 line) or sub-additivity (data lay under the line).

## Discussion

An acute 12-well plate phytotoxicity assay using isolated *H*. *ovalis* leaves was successfully developed and applied to the PSII herbicide Diuron. The assay applied PAM fluorometry (both Mini-PAM and I-PAM), which non-destructively measures change in the efficiency of PSII and will be useful to rapidly and reliably screen exposure to existing and emerging PSII herbicides. Fluorescence images demonstrated that Diuron affected PSII across the entire leaf surface evenly and measurements were not affected by isolating leaves from the plant. The inhibition of ∆*F*/*F*_m_’ (but not *F*_v_/*F*_m_) by Diuron in isolated *H*. *ovalis* leaves was identical to potted and hydroponic plants, following the optimum exposure duration of 24 h. The sensitivity of isolated *H*. *ovalis* leaves to Diuron was also consistent (i.e. IC_50_ and IC_10_ were similar) with other potted seagrasses (*Halodule uninervis* and *Zostera muelleri*) in a study that proposed revision of the environmental guidelines based on toxicity responses to four herbicides [18]. This similarity indicates that this rapid bioassay could be suitable for assessment of compliance with water quality guidelines. While the sensitivity of the assay was not influenced strongly by irradiance, high light levels did cause photo-oxidative stress in *H*. *ovalis*. Irradiance acted in an additive or sub-additive way with Diuron to impact on PSII thus highlighting the need for understanding how interacting environmental factors could influence screening procedures, as well as seagrass sensitivity to herbicides under variable environmental conditions.

### Optimising the assay

The main challenge in developing a well-plate phytotoxicity assay for isolated seagrass leaves was to ensure that the photosystems in the leaves were not degraded during the exposure period and that the exposure was sufficiently long for the leaves to reach steady-state response to the herbicide. The integrity of the leaves was confirmed in two related ways. Firstly, fluorescence images across the leaf surface using I-PAM showed that in uncontaminated seawater there was little difference in fluorescence response over the 24 h (Fig. 1). This was quantified by measuring the photosynthetic efficiency (*F*_v_/*F*_m_) in solvent controls and confirming there was <3% decrease over the exposure period. The fluorescence images also demonstrated that Diuron was acting on PSII evenly across leaves. Previous studies have shown very fine-scale differences in absorptivity across seagrass leaves [37] but this was less evident for ∆*F*/*F*_m_’ (which is minimised as it is based on a ratio of fluorescence emissions). Ralph et al. [37] also demonstrated differences in leaves of different ages with older leaves and the tips of leaves often exhibiting lower ∆*F*/*F*_m_’ values due to either chronic oxidative damage or differences in light adaptation over the length of a leaf. In the current study these differences were minimised by choosing young leaves and pre-screening for leaves with high photosynthetic efficiencies. Importantly the images demonstrated that the herbicide did not flood through the vascular system but entered the leaf evenly across the semi-permeable cell walls. This is consistent with a previous study which demonstrated that the PSII herbicide Atrazine was preferentially transported through the leaves rather than via the root-rhizome complex [38]. The uptake of Diuron was relatively rapid and steady state inhibition of ∆*F*/*F*_m_’ by ~12 h was consistent between the isolated leaf well-plate format and potted *H*. *uninervis* and *Z*. *muelleri* [18]. An optimum exposure was considered somewhere between 12 and 24 h when the herbicide had caused maximum toxicity, but the photosystems of leaves had not appreciably deteriorated. Our previous study using potted *H*. *uninervis* and *Z*. *muelleri* showed no additional inhibition of ∆*F*/*F*_m_’ or *F*_v_/*F*_m_ by Diuron between 24 and 72 h [18].

### Sensitivity in comparison with intact plants and studies

The inhibition of photosynthetic activity (∆*F*/*F*_m_’) by Diuron in isolated leaves using the 12-well plates was identical to inhibition in intact plants (both hydroponic and potted). The narrow range of IC_50_s (Table 1, 3.0–3.5 μg l^-1^) demonstrated not only the consistency of the method with larger, more time-consuming alternatives, but also supported our case that the isolated leaves are a valid alternative for acute phytotoxicity tests and supported previous studies that herbicide toxicity in seagrass is via leaf, not root-complex uptake [38]. The IC_10_ and IC_50_ values for ∆*F*/*F*_m_’ in isolated *H*. *ovalis* leaves were also similar to those reported for the effects of Diuron on ∆*F*/*F*_m_’ in potted *H*. *uninervis* and *Z*. *muelleri* over 24 and 72 h [18]. Although statistically different for ∆*F*/*F*_m_’, there was good agreement in assay results for the measurement of inhibition of leaves using the two PAM instruments (Table 1), which demonstrates that the 12-well plate technique is equally valid for use with a Mini-PAM or Diving-PAM (operationally the same as a Mini-PAM) if an I-PAM is not available. Although there was very good agreement of inhibition of ∆*F*/*F*_m_’ between well plate, potted and hydroponic methods, the horizontal leaves in well plates were more sensitive to the combination of herbicide and light as demonstrated by the lower *F*_v_/*F*_m_ in comparison with potted and hydroponic leaves (Table 1). We had determined the most reliable way of ensuring similar photosynthetic activity of the controls in horizontal isolated leaves and intact plants was to adjust the irradiance so that ∆*F*/*F*_m_’ was similar for both cases (100 and 280 μmol photons m^-2^s^-1^ respectively). However, it seems that the horizontal leaves at 100 μmol photons m^-2^s^-1^ may have been under greater photooxidative pressure (lower *F*_v_/*F*_m_) using these conditions [30]. This result does not affect the utility of this assay (and has no effect on the inhibition of ∆*F*/*F*_m_’), but highlights the influence of irradiance on PSII by herbicides and may differ between isolated leaves and intact plants.

### Consistency of endpoints under different irradiance

As discussed above, the sensitivity of aquatic plants to PSII herbicides can be influenced by irradiance. Photosynthetic activity increases with irradiance (light-limited phase) until saturating irradiances are reached and ∆*F*/*F*_m_’ values are reduced as more PSII reaction centres become inactive (photoinhibited phase) (Fig. 6A), and energy is dissipated by fluorescence and non-photochemical quenching [34]. Diuron and other PSII inhibitors also decrease the capacity of PSII by blocking electron transport [22,23,39] and ∆*F*/*F*_m_’ is further reduced (Fig. 6A). Indeed the model of Independent Action (Fig. 7A) demonstrated that the effect of irradiance and Diuron on photosynthetic activity were largely additive across the conditions tested. In the present study there was little difference in IC_50_ values for ∆*F*/*F*_m_’ (2.2–3.5 μg l^-1^) under different irradiances (when inhibition was calculated against the ∆*F*/*F*_m_’ of control leaves at the corresponding irradiance). This result shows that although the irradiance spanned an order of magnitude that the relative inhibition of ∆*F*/*F*_m_’ was not appreciably influenced, further highlighting the robust nature of this endpoint.

### Cumulative impacts of Diuron with high irradiance

High irradiance has the potential to cause photo-oxidative damage to PSII and the rapid light curve indicated 400 μmol photons m^-2^ s^-1^ far exceeded the photosynthetic capacity of these leaves [34] and would have caused stress to the photosystems. This was demonstrated by the high level of inhibition seen in *F*_v_/*F*_m_ by 400 μmol photons m^-2^ s^-1^ relative to control values at the lowest illumination, even at low herbicide concentrations (Fig. 6B). The measured inhibition of *F*_v_/*F*_m_ by combinations of high irradiance and Diuron was generally additive (close to the expected inhibition according to the IA model, Fig. 6B). However, both combinations of light and Diuron that most exceeded a 1:1 ratio (indicating a synergistic effect) were high irradiance treatments. Generally combinations of Diuron and lower light yielded combined impacts on *F*_v_/*F*_m_ that were sub-additive. Regardless of the scale of interaction the combined impacts of Diuron and high irradiance was to increase the impact on PSII (Fig. 6B). This highlights the need for carefully controlled and consistent environmental conditions during future screening, particularly in relation to compliance assessment. This finding that high irradiance increases the sensitivity of seagrass to herbicides adds to the growing body of evidence that herbicides increase the vulnerability of tropical species, such as corals [40] and foraminifera [35], to other pressures such as climate-related high sea surface temperatures. While no other multiple-stress experiments have been performed on seagrass in concert with herbicide exposure, seagrasses are vulnerable (especially photosynthetic efficiency) to changing environmental conditions including osmotic stress [41], desiccation [42,43] thermal stress [41,44] and low light [45]. Herbicides could exacerbate sensitivity of seagrasses to these other environmental factors, and more studies are needed to quantify how they may be protected from climate pressures by improving water quality, and reducing herbicide residue accumulation in the coastal habitats.

### Application of the assay

While fluorescence well-plate assays have been used widely and effectively to study the impacts of PSII herbicides on microalgae [26,46], only one previous study has attempted to apply I-PAM fluorometry to investigate herbicide toxicity in multicellular aquatic plants in a miniature well plate system. Küster and Altenburger [25] carried out a similar toxicological experiment uisng macrophyte and macroalgae thallus samples exposed to PSII herbicides in 24-well plates over 24 h. Their results are consistent with our study in demonstrating that the utility and convenience by which macrophyte leaves can be used as relevant biomaterial for acute assasys, as long as the extent of herbicide uptake and leaf heath have been assessed as appropriate and compare well with intact plants. The *H*. *ovalis* well-plate assay was able to detect effects on photosynthetic capacity at concentrations lower than environmental guidelines for 90% species protection within the GBR [47]. These results closely correspond to those presented in previous acute toxicity studies of other tropical seagrass species common along the GBR [18]. The high sensitivity of the well-plate assay should enable its application as a biomonitoring technique for existing and emerging PSII herbicides in natural waters [48]. *H*. *ovalis* has proven to be a suitable assay species [10,24,49], as its ovate leaves are easily isolated with minimal damage, the leaves are small and fit within a micro-assay setup and it is a ubiquitous species, occurring throughout the Indo-Pacific in coastal and estuarine habitats which are at the forefront of exposure to runoff containing herbicides. Similar assays could be developed for other large-leafed species but care should be taken to minimise injury to leaves by cutting and validation that preferential uptake of herbicide via cutting wounds is negligible. This assay for acute effects of PSII herbicides on photosynthetic capacity (∆*F*/*F*_m_^’^) and maximum quantum yield (*F*_v_/*F*_m_) to PSII in seagrass is likely to be applicable to a variety of PSII herbicides under a broad range of conditions such as reduced salinity and temperature extremes. It does not rely on elaborate experimental aquarium systems to maintain potted plants and takes far less time, resources and biological material to deliver reliable ecotoxicology data. Application of this assay has the potential to provide management agencies and regulators with considerably more toxicological data for the development of improved risk assessments and water quality guidelines.
